# The Reliability and Validity of an Assessment Tool for Developmental Dyslexia in Chinese Children

**DOI:** 10.3390/ijerph17103660

**Published:** 2020-05-22

**Authors:** Anyan Huang, Kusheng Wu, Anna Li, Xuanzhi Zhang, Yuhang Lin, Yanhong Huang

**Affiliations:** 1 Mental Health Center, Shantou University Medical College, North Taishan Road, Shantou 515065, China; 19ayhuang@stu.edu.cn (A.H.); zhangxuanzhi2018@163.com (X.Z.); 18yxlin@stu.edu.cn (Y.L.); 2 Department of Preventive Medicine, Shantou University Medical College, Shantou 515041, China; kswu@stu.edu.cn (K.W.); st_lianna@163.com (A.L.)

**Keywords:** dyslexia, reliability, validity, confirmatory factor analysis, auxiliary diagnosis

## Abstract

Developmental dyslexia (DD) is a common neurobehavioral disorder in children. It refers to the phenomenon in which children with normal intelligence lag significantly behind their peers in reading ability. In China, there is no unified standard for the assessment of dyslexia due to the use of simplified and traditional Chinese characters in different regions. This study was aimed at analyzing the reliability and validity of the self-developed Chinese dyslexia assessment tool named Chinese Reading Ability Test (CRAT), which was suitable for students of grade 3 to 5 in primary school. We randomly selected three primary schools in Shantou city of China, including two in the central district and one in the surrounding district. A total of 1492 students of grades 3 through 5 were recruited. We assessed the reliability of CRAT by test–retest reliability and internal consistency. The validity assessment was realized by discriminant validity, content validity and confirmatory factor analysis (CFA). For reliability, the test–retest correlation coefficient of the total score of the CRAT was 0.671. The difference between the test–retest was not statistically significant. The Cronbach’s alpha coefficient of the CRAT was 0.75. For validity, the correlation coefficient between the score of each subscale and the total score of the scale ranged from 0.29 to 0.73. The indexes of the three structural equation models all meet the standard (*p* > 0.05, χ^2^/df < 2.00, RMSEA < 0.05, GFI > 0.90, AGFI > 0.90, NFI > 0.90, CFI > 0.90 and IFI > 0.90). The fitting effects of the models were good. The CRAT has sufficient reliability and validity which could be used for the assessment and auxiliary diagnosis of Chinese Dyslexia in primary school students of grade 3 to 5.

## 1. Introduction

Developmental dyslexia (DD) is a persistent reading disorder characterized by inaccurate or slow or effortful reading and also by poor spelling [1]. These difficulties are often “unexpected” in view of the child’s other cognitive abilities (i.e., typical general intelligence) and exist despite the provision of adequate formal classroom instruction. At the explanatory level, dyslexia typically results from a deficit in the phonological component of language [2]. The occurrence of dyslexia is universal. It occurs in all languages [3], even though the consistency in which phonology is represented in the orthography varies and has a major effect in the development of reading [4]. Although our understanding of dyslexia has grown in the past decades, the concept and research of dyslexia is still in its infancy in many non-English speaking countries around the world [5]. Although the educational and sociocultural resources of children with dyslexia are not different from those of ordinary children, their reading ability is lower than the expected level of the current age [6]. Some studies also found that children with dyslexia have problems with reading comprehension and social skills, which may affect their academic performance and income during adulthood [7,8]. According to some studies, the incidence of dyslexia in school-age children in English-speaking countries ranges from 5% to 10% [9,10]. Chinese society is improving its understanding of specific learning difficulties such as dyslexia, but there is still a relative lack of understanding of dyslexia, especially in mainland China. Chinese is a nonalphabetic language. Although the logography and orthography in Chinese characters make it easier to grasp meaning when compared alphabetic languages, the incidence of dyslexia in China is not low and the incidence rate is very close to that of other English-speaking countries [11]. According to most research findings in China, the incidence of dyslexia in China is about 3% to 10% [11,12,13].

So far, there has been a lot of DD related researches in China. However, the assessment of dyslexia in China is quite confusing. One of the reasons is that the complexity of the Chinese they use is different. In mainland China, simplified Chinese is usually studied and used, while in Hong Kong, Macau and Taiwan, children learn how to read in traditional Chinese. Compared with simplified characters, traditional characters have more strokes [14]. Moreover, in most studies of children with dyslexia, the selection criteria for children with dyslexia were set by the investigators themselves. There is not any single standardized test for dyslexia confirmation in China [15]. Therefore, a more standard dyslexia assessment tool should be established as soon as possible to assist in clinical diagnosis. In this way, the targeted interventions for children with dyslexia can be implemented in a timely manner. Dyslexia is not a single cognitive deficit, but multiple deficits in a series of perceptual and cognitive processes [16]. Frost suggested that as much as possible to develop a possible universal model of reading, the orthographic processing, phonological awareness and morphological awareness must be considered [17]. For Chinese, the most common language processing defects in dyslexia (in descending order) are rapid automatized naming (50% of children involved), orthographic awareness (39%), and phonological awareness (20%) [16,18]. Therefore, understanding the basic language processing skills essential to Chinese reading is the basis for the establishment of standardized and universally applicable Chinese dyslexia assessment tools. When we understand these language processing skills, we can use these skill tests to assess children who may have dyslexia.

Phonological awareness refers to the ability to identify and manipulate units of sound. It is the main indicator of cross-linguistic dyslexia [19]. In general, a deficit in processing phonology is generally considered an important risk factor for children with dyslexia [20]. Shu et al found that morphological awareness may be a core theoretical construct necessary to explain variability in reading Chinese [11]. In particular, morphological awareness is uniquely important for early Chinese character recognition [21]. Rapid automatized naming (RAN) measures acts as a microcosm of the reading system, providing an index of one’s abilities to integrate multiple neural processes [22]. RAN usually refers to any rapid automatized naming task or process. It is worth noting that defects in vocabulary access and retrieval are found in children with dyslexia, which are usually measured by RAN [23,24]. Orthographic processing skill refers to children’s ability to pick out false characters that violate the structure of Chinese characters and to recognize the typical position of the radical in Chinese characters. A study using the event-related potential (ERP) method demonstrated that the brain response of dyslexics lacks the conditions to distinguish between false and nonwords, suggesting that dyslexics cannot use orthographic processing rules to identify incorrectly constructed characters [25]. In general, research over the past decade focused on the importance of phonological awareness, orthographic awareness, morphological awareness and RAN in learning to read Chinese words [23,26,27]. However, little is known about the reading comprehension of Chinese school-age children. According to the Simple View of Reading [28], reading consists of two basic components: word decoding and reading comprehension. Fuchs et al. found that word reading fluency, accuracy and reading comprehension are interrelated, because effective word recognition frees processing resources to focus on comprehension [29]. Moreover, reasoning skills may be especially useful for reading comprehension [30]. Therefore, it is particularly important to combine language processing skills with reading ability to assess dyslexia.

Based on the above language processing skills and the reading ability, we developed an assessment tool of DD which was named the Chinese Reading Ability Test (CRAT). It is suitable for Chinese primary school students in grade 3 to 5 who speak simplified Chinese as their mother tongue. The purpose of present study is to analyze the reliability and validity of the self-developed CRAT.

## 2. Materials and Methods

### 2.1. Participants

The method of stratified random cluster sampling was used to recruiting primary schools in Shantou, China. We randomly selected one public primary school in each of the two central areas of the town and one public primary school in the surrounding rural areas. The process of randomization was performed by the random number generation program of SPSS 24.0 (IBM Corporation, Armonk, NY, USA). We followed the including criteria as: (1) the participants didn’t have a history of neurological diseases or psychiatric disorders; (2) the participants had normal or corrected-to-normal vision; (3) all of the participants had normal IQ; (4) all of the participants were permanent residents of Shantou having lived there for more than half a year.

### 2.2. Procedures

Prior to the survey, the research group carried out lectures in each school to introduce the relevant knowledge of DD, the research purpose of this project, the investigation process and matters needing attention, etc. The systematic training was conducted to investigators before investigation. Both the respondents and their guardians agreed to participate in the survey and the guardians signed an informed consent before investigations.

**Figure 1:**
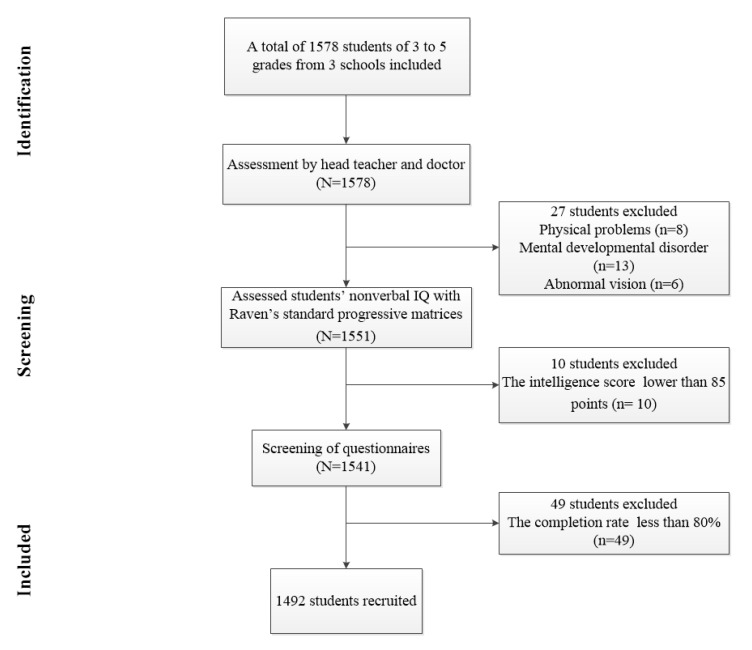
Flow chart of the participant selection process.

A total of 1578 students were included. First of all, we spoke to the head teacher of the corresponding classes to know about the physical condition of the students, excluding the students with poor reading and writing ability due to physical problems, mental development disorders and other problems (*n* = 21). At the same time, we also excluded students with abnormal vision or vision still abnormal after correction via the assessment of a professional doctor (*n* = 6). Secondly, we assessed students’ nonverbal IQ with Raven’s standard progressive matrices that were commonly used to estimate nonverbal reasoning ability in previous studies [11,31]. According to the intelligence level of the test, when the intelligence score of the respondents is lower than 85 points, it indicates that their intelligence may be defective. Therefore, participants with intelligence scores below 85 were excluded from this study (*n* = 10). Thirdly, after the test, the questionnaire was collected uniformly. Before the data analysis, the finished questionnaires were screened. The scale with a completion rate of less than 80% was removed (*n* = 49). Finally, a total of 1492 students in grades 3 through 5 were recruited. The flow chart of participant selection process is shown in Figure 1. 

Referring to the study [32], the three-stage survey of screening–interview–diagnosis was used in our present study. In the screening stage, we first used the Raven’s standard progressive matrices and the Character Recognition Measure and Assessment Scale for Primary School Children. According to the study in mainland China, children diagnosed with dyslexia performed at least one standard deviation (SD) below the mean for their grade level on literacy tests [33]. Therefore, in the present study, children who have a normal IQ (an intelligence score greater than or equal to 85) and character recognition scores of at least one SD lower than normal were selected. In the interview stage, the psychiatrist conducted a half structured interview with the head teachers of the students. The purpose of this stage was to understand whether the selected students had some related clinical symptoms of dyslexia, as well as to obtain information regarding the students’ usual Chinese scores, physical conditions, learning attitude, etc., and excluding the physical problems, mental development disorders and other problems which can cause poor literacy. In the final diagnosis stage, two experienced psychiatrists discussed the results of the interview phase and followed the diagnostic criteria in DSM-5 to make the final diagnosis. When the diagnosis was controversial, a third psychiatrist joined the discussion and confirmed the diagnosis. We selected the diagnosed dyslexia students from school A for discriminant validity analysis. A total of 57 students diagnosed with dyslexia were selected. Meanwhile, 57 students (in school A) who were matched with the dyslexia group in grade, gender and age with normal intelligence and above average scores on the literacy tests were selected as the control group.

Two weeks after the first test, we used the cluster random sampling method to retest a random class of students from the selected school (school A) (n = 45). Among the recruited students, six of them did not finish the retest due to absence and other reasons.

### 2.3. Instruments

#### 2.3.1. Raven’s Standard Progressive Matrices

The test consists of five units, each with 12 items, a total of 60 items [34]. Each item was made up of a target visual matrix with a missing piece. Students were required to pick, from six to eight alternatives, the best parts to complete the target matrix. The maximum score was 60, and Cronbach’s α was 0.91.

#### 2.3.2. Character Recognition Measure and Assessment Scale for Primary School Children

The scale consists of 210 characters and it was divided into ten groups based on the reading-difficulty level [35]. The participants were requested to write down a compound word based on a constituent morpheme provided on the sheet. Their performance was measured by the total number of correct characters that they could utilize in character compounds. The mean and standard deviation of the performance of character recognition were calculated for each grade.

#### 2.3.3. Chinese Reading Ability Test (CRAT)

The CRAT was established to assist in the clinical diagnosis of Chinese dyslexia in grades 3 to 5 students at the primary school in China. It was composed of five subscales, namely, the phonological awareness subscale, the morphological awareness subscale, the rapid automatized naming subscale, the orthographic awareness subscale, and the reading ability subscale.

- Phonological Awareness Subscale

The phonological awareness subscale was based on the study published by Shu et al. [11]. The test was a recording that consisted of three subtests measuring onset, rhyme, and tone, respectively. Each subtest had 12 items and each item had 4 options. There were 36 items altogether. The experimenter of audio pronounced single-syllable real word two times and the children were later requested to select the one syllable that was different from the other three. In this test, 1 point was scored for an item, and there was a total of 32 points. We record the scores of tone, onset, and rhyme as Sd1, Ss1, and Sy1, respectively. The total score for the phonological awareness test was recorded as PA.

- Morphological Awareness Subscale

According to the study [36], there are five Chinese compounding structures: subordinate, coordinative, subject–predicate, verb–object, and verb/adjective–complement. Therefore, the evaluation of morphological awareness in the present study includes the compounding structures as described, with a total of ten groups, each with eight characters arranged in two columns, and each column consisting of four characters. The children were required to combine each character of the first column of each group with one of the characters in the second column to form a compound, and each character has only one chance to be combined into one compound. Each group of characters will eventually form four compounds. In this test, we recorded the student’s answer time as morphological awareness test time (recorded as T2). Four compounds of each group were correctly paired to score one point, and ten groups of compounds all correctly formed scored ten points. We recorded this test score as (S2). This allowed us to obtain the combination of S2/T2—the morphological awareness test efficiency (S_t_2). The total score for the phonological awareness test was recorded as MA.

- Rapid Automatized Naming Subscale

Analogous to a study [37], the children were given a piece of paper with 40 numbers in this task. The paper shows five numbers (9, 6, 4, 2, and 7) replicated eight times in a different order. To ensure that children were well aware of the stimulus, we required them to name them independently in advance. After that, children were asked to name all numbers from left to right, top to bottom, twice, as quickly and accurately as possible. The time was recorded with a stopwatch. We recorded the naming time and the total number of verified named numbers twice, respectively. The average of the twice-named time (recorded as T3) and the average of correctly twice-named numbers (recorded as S3) were calculated. This allowed us to obtain the combination of S3/T3—the naming efficiency (recorded as S_t_3). The total score for the rapid automatized naming was recorded as RAN.

- Orthographic Awareness Subscale

The orthographic awareness subscale included two tests, the naming noncharacter recognition test and the radical position test. The noncharacter recognition test was used to measure the children’s knowledge of Chinese character structure and the sensitivity of students to distinguish between characters and noncharacters. There were ten left–right and up–down structured rare characters and ten noncharacters. The rare characters might have looked unfamiliar to the children, but they were real Chinese characters that conformed to the legal character structure. Noncharacters were characterized by extra strokes or missing strokes. The children were required to choose which rare characters are and which noncharacters are. There were ten rare characters and ten noncharacters in the test. A score of 20 points would represent all problems answered correctly. The total score for the noncharacter recognition was recorded as S_s_4.

The radical position test measured the children’s knowledge of the positional regularity of Chinese radicals. 12 semantic and phonetic radicals were chosen as stimuli. The children were asked to indicate the legal position of each radical out of four choices (left, right, top, and bottom). There were 12 multiple choice questions. The total score was 20 points when all the answers were correct. The points scored by correctly identifying the radical position of character (recorded as s4) and the time taken to identify the radical position of character (recorded as t4) was measured. This allowed us to obtain s4/ t4—the radical position awareness test efficiency (recorded as St4). The total score for the orthographic awareness was recorded as OA. These two tests were based on the test methods used in the study which is published by Ho et al. [16].

- Reading Ability Subscale

According to some studies [38,39], reading comprehension skills are measured by four comprehension processes in the International Reading Literacy Study (PIRLS). These four comprehension processes were: (1) focus on and retrieved noted information and ideas; (2) make straightforward inferences; (3) interpret and integrate ideas or information and (4) examine and evaluation content, language, and textual elements. Therefore, the test in the present study was designed to measure primary school students’ word reading fluency, accuracy, and Chinese reading comprehension skills. This reading test was adapted from a short narrative essay on the teaching test syllabus. The essay had a total of 295 characters, and each character had a syllable on it. The essay was followed by three test questions. These three questions were the cloze test, verbal question, and sentence. They examined the full-text reading comprehension, integration of ideas or information and evaluation of content and syntactic skills, respectively. The specific test process was: First, we asked students to read the entire essay and recorded the number of characters correctly read by the students when reading the essay for a minute, which provided us with N1—reading fluency. The number of characters correctly read when reading the full-text was recorded as N2, and the time to read the full text was recorded as T5. This provided us with N2/T5—reading accuracy (recorded as N_t_5). Next, the students were asked to complete three questions on the essay and time spent on providing answers was recorded. The scores of these three questions were recorded as s5, s6, s7. The maximum point for each question was 5, with a total of 15 points. The total score of these questions was the total score of reading comprehension, recorded as S_a_. The test prevented dyslexic readers and poor readers from compensating for their written word recognition difficulties by using contextual information. The total score for the reading test was recorded as RA.

### 2.4. Statistical Methods

The database was built using Epidata 3.1 (Jens M. Lauritsen, Odense, Denmark) independently by two professionals. Data after verification were exported to SPSS 24.0 (IBM Corporation, Armonk, NY, USA) for data analysis. The CFA was performed using AMOS, version 21.0 (IBM Corporation, Armonk, NY, USA). In the present study, the missing value of the test sample was replaced by the sequence mean of the corresponding variable. Since the collected data did not follow a normal distribution, it was described by the median and the quartile range, which were expressed as M (P_25_ ~ P_75_). At the same time, since some of the tests in this study involve time and score, according to the definition of the study, the higher the score and the shorter the time spent on the test, the better the mastery of the skill. Therefore, in order to make the direction of the interpretation of the research results consistent—that is, the larger the measurement score, the better the performance of the children—, the measurement time and the score need to be converted into efficiency (i.e., equal to the score/time). Correlation analysis between variables was performed using Spearman correlation analysis.

## 3. Results

### 3.1. Reliability Statistics

#### 3.1.1. Test–retest Reliability

Spearman correlation analysis was conducted on the test and retest, and the results showed that test–retest reliability of each subscale and total score was between 0.442 and 0.790. There was no significant difference in the results of subscale scores and total scores before and after the measurements by nonparametric test (all *p* > 0.05). All of the subscales showed moderate to good reliability. The results are shown in Table 1.

#### 3.1.2. Internal Consistency Reliability

Spearman correlation coefficients of each subscale and total score were measured respectively. The internal consistency of each subscale and total score was estimated, so as to assess the consistency among all items in the scale. In this study, nonphonological awareness (NPA) is the sum of other language processing skills besides phonological awareness. Spearman correlation coefficient of each subscale was between 0.399 and 0.448 and the average correlation coefficient was 0.422. The correlation coefficient between subscale and total score ranged from 0.684 to 0.869 and the average correlation coefficient was 0.759. The Cronbach’s Alpha coefficient of internal consistency index of each subscale ranged from 0.42 to 0.76. The Cronbach’s Alpha coefficient of the total scale was 0.75. The results are shown in Table 2.

### 3.2. Validity Statistics

#### 3.2.1. Discriminant Validity

Discriminant validity refers to that the scale can distinguish two known groups of different people (such as the dyslexic group and the control group in the present study). The purpose of this test is to analyze the external validity of the scale. From Table 3, we can see that the scores of Sd1, Ss1, Sy1, S_t_2, S_t_3, S_s_4, S_t_4, N1, N_t_5, S_a_ and the total score in the dyslexic group were higher than those in the control group, that is, the difference between the dyslexic group and the control group was significant (*p* < 0.05). The above results show that the scale has good discriminant validity.

#### 3.2.2. Content Validity

Content validity refers to whether the selected test item can represent the contents to be tested. In this study, the content validity of the CRAT was measured by calculating the correlation coefficients between each subtest, between each subtest and the corresponding subscale, and between each subscale and the total scale. The correlation coefficient of each test of the PA subscale ranged from 0.44 to 0.46. The correlation coefficients between the scores of each test of the PA subscale and the total score of the PA subscale ranged from 0.73 to 0.81. The correlation coefficient of each test of the NPA subscale ranged from 0.11 to 0.31 and the correlation coefficients between the scores of each test of the NPA subscale and the total score of the NPA subscale ranged from 0.43 to 0.77. The correlation coefficient ranged from 0.10 to 0.25 between each test of RA subscale. The correlation coefficient between each test scores of the RA subscale and the total score of the RA subscale ranged from 0.48 to 0.71. The correlation coefficients between each subscale and the total scale ranged from 0.29 to 0.73.

#### 3.2.3. Confirmatory Factor Analyses (CFA) of the CRAT

**Figure 2:**
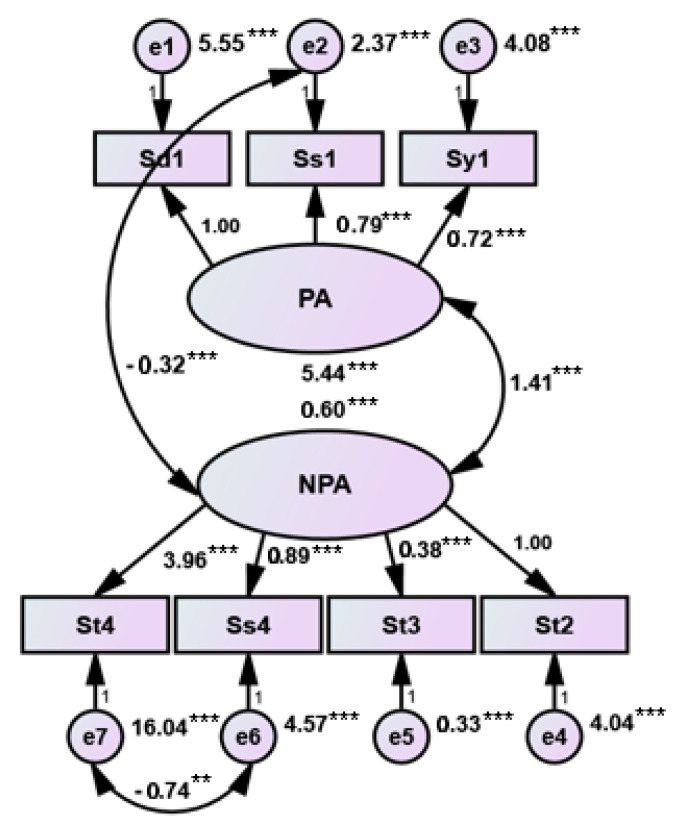
Cognitive skill measurement model (Note: ** *p* < 0.01. *** *p* < 0.001, two-tailed).

**Figure 3:**
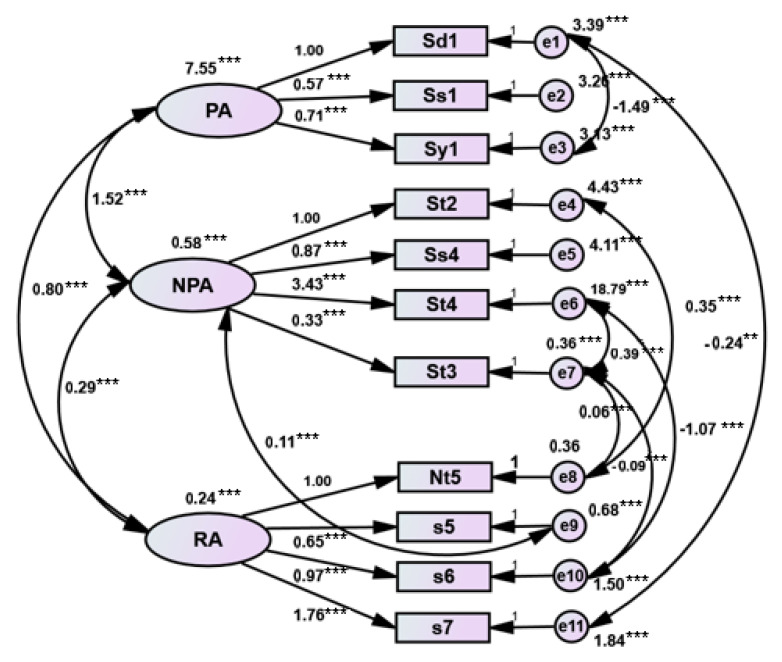
Cognitive skills and reading measurement models (Note: ** *p* < 0.01. *** *p* < 0.001, two-tailed).

**Figure 4:**
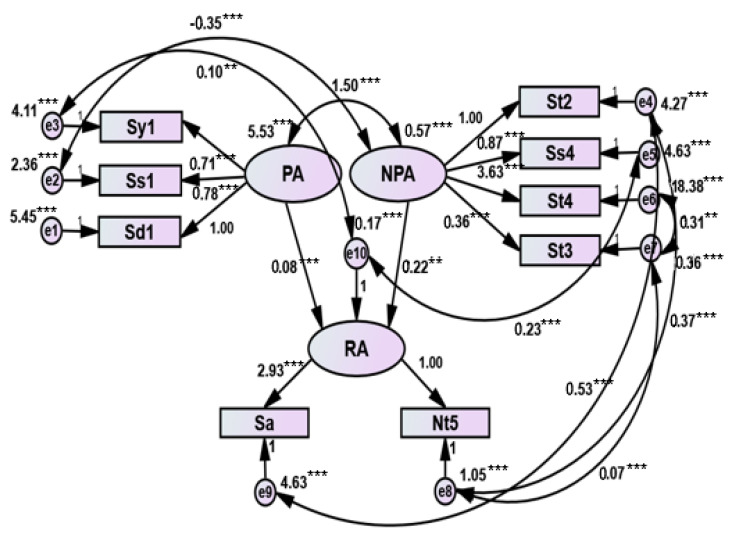
Cognitive skills and reading structural equation models (Note: ** *p* < 0.01. *** *p* < 0.001, two-tailed).

Structural validity refers to whether the multi-index measurement of objective things has an ideal professional structure. The evaluation of structural validity usually has no “golden standard” or expert opinions to refer to. It is necessary to collect a certain amount of actual survey data and use a statistical analysis method for analysis and evaluation. In this study, CFA was used to verify whether the structure of the CRAT was professionally ideal, and the structural equation model was further studied. Because the data collected in this study are of non-normal distribution, the model is fitted by asymptotic distribution free (ADF) which also known as weighted least squares (WLS) in the robust estimation method. The measurement model of language processing, the measurement model of language processing and reading, and the structural equation model of language processing and reading of the CRAT fit well with the actual observation data. According to the fitting results of the scale structure model which are shown in Table 4, it can be observed that these three models have high usability. The models are shown in Figure 2, Figure 3 and Figure 4 separately.

## 4. Discussion

By referring to literatures and previous work, we developed the CRAT to assess DD in Chinese primary school students with simplified Chinese as their mother tongue. Some studies found that Chinese lexical compounding awareness which is understood as “how morphemes can be combined sensibly in the Chinese language” was able to predict uniquely Chinese character reading and vocabulary knowledge in the primary grades [40]. Therefore, in some studies, homophone awareness and/or lexical compounding was/were utilized to distinguish people with and without dyslexia [11,41]. Moreover, morphological awareness has also contributed uniquely to Chinese character writing, reading fluency, and reading comprehension concurrently and longitudinally [39,42,43]. In China, it was clearly indicated that children with dyslexia consistently struggled in RAN tasks [44]. Ho et al. noted that orthographic processing skills have been a considerable issue of investigations of Chinese developmental dyslexia [18]. We combined the above four measures of language processing skills with additional measures of reading ability to form our assessment tool, CRAT. At the same time, we consulted primary school Chinese teachers with rich teaching experience and conducted case tests in outpatients of Shantou University Mental Health Center to repeatedly modify and improve the CRAT. In order to verify the authenticity and dependability of the CRAT, we conducted the reliability and validity analysis. The results of this study showed that the self-developed CRAT had good reliability and validity.

The significance of test–retest reliability is the stability of the scale. There was no statistically significant difference between the two measurement results of subscales and total scale (all *p* > 0.05). The specific reason for the general reliability of some subscales may be caused by the children’s improper control of the test time. The small sample for test–retest might also have accounted for the lower reliability. However, according to the study, an adequate value above 0.60 and 0.70 for test–retest reliability is better [45]. It is indicating that the stability of the CRAT is still acceptable. For the internal consistency reliability, all of the correlation coefficients have significant significance, which can be explained to some extent that all of the participants focus on the unified psychological structure or characteristics. The Cronbach’s alpha value was used to determine internal consistency of the scale. Lance et al. concluded that an acceptable cut-off value of total scale is 0.70 [46]. In the present study, the Cronbach’s alpha coefficients of NPA and RA are below 0.6. The reason for the lower coefficients may be because some test questions are designed to be too simple or difficult to understand. The Cronbach’s alpha coefficients of PA are above 0.75. The Cronbach’s alpha coefficient of the total score is 0.75, which means that the internal consistency of the CRAT is acceptable [47].

Our results show that the discriminant validity and the content validity of the CRAT are good. It can be concluded that the validity of the CRAT is fundamentally in good condition. For the structural validity of the CRAT, CFA and structural equation model analysis were used to measure the validity. According to previous studies in China [37,39], we can see that there is a certain correlation among the variables of language processing skills and the indicator variables of language processing skills are also correlated with those of reading ability. Another study also showed that verbal working memory had a strong direct effect on the reading comprehension of Chinese children in grades 3 to 5 [48]. Moreover, discourse skills and syntactic skills were substantially correlated with Chinese reading comprehension in primary school students [49]. Therefore, in this study, the structural validity of CRAT was analyzed by three different measurement models, further evaluating the fitting degree of the structural equation model. According to the study published by Byrne [50], the results of the present study showed that the fitting indexes of the three structural models all met the standards (*p* > 0.05, χ^2^/df < 2.00, RMSEA < 0.05, GFI > 0.90, AGFI > 0.90, NFI > 0.90, CFI > 0.90, IFI > 0.90). It is indicated that the causal relationship between language processing skills (including PA and NPA) and reading ability was verified. The correlation also presented among the tests of language processing skills. From this we can draw the conclusion that the three structural models fit well. In summary, our results showed that CRAT has sufficient reliability and validity, and it can effectively assist in the clinical diagnosis of children with dyslexia.

Our study has some limitations. Some of the children may feel impatient because of the long time spent on the test. It was difficult to confirm whether children had fully understood the questions when the investigators explained the answer requirements to them, which may have cause inaccurate test results. In addition, the lesser participants included in the retest analysis may compromise reliability. Only using numbers as stimuli of RAN also lacks certain richness. Therefore, further improvement on more information and a more appropriate approach is still needed in the future practical application of CRAT.

## 5. Conclusions

The CRAT has satisfactory reliability and validity. The measurement model and structural equation model of the CRAT have good availability and the structure of these models reaches the ideal structure in the profession. Moreover, not only can the CRAT efficiently screen 3 to 5 grade school-aged children with suspected dyslexia in the crowd, but it can also be used for further analysis of the participants in the four dimensions of language processing skills including phonological awareness, morphological awareness, orthographic awareness, rapid automatized naming as well as reading. CRAT can also provide theoretical basis and guidance for the development of follow-up individualized intervention measures and rehabilitation training programs for children with dyslexia.

## Figures and Tables

**Table 1:** Test–retest reliability of each subscale and total score.

	Test Score M (P_25_ ~ P_75_)	Retest Score M (P_25_ ~ P_75_)	*R*
Phonological Awareness	24.00 (21.00 ~ 28.00)	25.00 (19.00 ~ 27.00)	0.599 **
Morphological Awareness	3.28 (2.80 ~ 3.93)	4.00(2.89 ~ 4.88)	0.790 **
RAN	2.21 (1.98 ~ 2.55)	2.23(1.93 ~ 2.51)	0.757 **
Orthographic Awareness	24.82 (21.43 ~ 26.24)	25.35(23.27 ~ 29.96)	0.442 **
Reading Ability	12.48 (11.19 ~ 14.86)	11.92(9.77 ~ 14.04)	0.652 **
Total Score	80.45 (67.20 ~ 87.64)	84.22(72.05 ~ 90.41)	0.671 **

** *p* < 0.01; RAN: rapid automatized naming; R: spearman coefficient; the test score and retest score are described by the median and the quartile range, expressed as M (P_25_ ~ P_75_).

**Table 2:** Internal consistency reliability of each subscale and total score.

Subscale	PA	NPA	RA	Total	Cronbach’s Alpha
PA	1.000	-	-	0.869 **	0.76
NPA	0.448 **	1.000	-	0.725 **	0.42
RA	0.399 **	0.418 **	1.000	0.684 **	0.50
Total	0.869 **	0.725 **	0.684 **	1.000	0.75

** *p* < 0.01; PA: phonological awareness; NPA: the sum score of the morphological awareness, rapid automatized naming and orthographic awareness; RA: reading ability.

**Table 3:** Differences of each subscale in the Chinese Reading Ability Test (CRAT) test between the dyslexia group and the control group.

	Dyslexia (*n* = 57)	Control (*n* = 57)	*Z* *	*p*
**PA**				
Sd1	9.00 (5.50 ~ 11.00)	12.00 (10.00 ~ 12.00)	−4.65	<0.001
Ss1	9.00 (7.00 ~ 10.00)	10.00 (9.00 ~ 11.00)	−4.52	<0.001
Sy1	8.00 (5.00 ~ 9.00)	10.00 (8.00 ~ 11.00)	−4.43	<0.001
**MA**				
T2	162.00 (147.50 ~ 186.50)	135.00 (120.00 ~ 160.00)	−4.07	<0.001
S2	9.00 (9.00 ~ 10.00)	10.00 (10.00 ~ 10.00)	−5.05	<0.001
S_t_2	3.41 (2.90 ~ 3.99)	4.44 (3.80 ~ 4.87)	−5.01	<0.001
**RAN**				
T3	16.50 (14.50 ~ 19.25)	13.50 (12.00 ~ 15.75)	−4.67	<0.001
S3	40.00 (39.50 ~ 40.00)	40.00 (40.00 ~ 40.00)	−1.48	>0.05
S_t_3	2.35 (2.07 ~ 2.76)	2.96 (2.54 ~ 3.29)	−4.75	<0.001
**OA**				
S_s_4	15.00 (14.00 ~ 16.50)	17.00 (16.00 ~ 18.00)	−3.94	<0.001
t4	32.00 (27.50 ~ 40.00)	30.00 (25.00 ~ 34.00)	−2.33	<0.05
s4	11.00 (10.00 ~ 11.00)	11.00 (11.00 ~ 12.00)	−3.06	<0.001
S_t_4	10.00 (7.90 ~ 11.69)	11.79 (9.55 ~ 14.07)	−2.87	<0.001
**RA**				
N1	180.00 (152.00 ~ 211.00)	221.00 (200.50 ~ 247.50)	−4.88	<0.001
T5	100.00 (80.50 ~ 118.50)	81.00 (70.50 ~ 86.00)	−4.85	<0.001
N2	290.00 (288.00 ~ 292.00)	293.00 (291.00 ~ 294.00)	−4.66	<0.001
N_t_5	2.94 (2.42 ~ 3.57)	3.64 (3.39 ~ 4.17)	−4.96	<0.001
S_a_	10.00 (8.00 ~ 12.00)	11.00 (10.00 ~ 12.00)	−2.16	<0.005
s5	2.00 (2.00 ~ 3.00)	2.00 (2.00 ~ 3.50)	−1.07	>0.05
s6	4.00 (3.00 ~ 5.00)	4.00 (3.50 ~ 5.00)	−1.75	>0.05
s7	4.00 (2.00 ~ 4.00)	4.00 (3.00 ~ 5.00)	−2.17	<0.05

* nonparametric assumptions; PA: phonological awareness; MA: morphological awareness; RAN: rapid automatized naming; OA: orthographic awareness; RA: reading ability; the test items represented by each abbreviation can be found in the materials and methods; the test score of dyslexia group and control group are described by the median and the quartile range, expressed as M (P_25_ ~ P_75_).

**Table 4:** Model fitting index.

	*p*	χ^2^/df	GFI	RMSEA	AGFI	NFI	CFI	IFI
Model 1	0.307	1.163	0.995	0.011	0.987	0.975	0.996	0.996
Model 2	0.137	1.271	0.991	0.014	0.982	0.944	0.987	0.987
Model 3	0.517	0.947	0.995	0.000	0.988	0.975	1.000	1.001

Model 1: Cognitive skill measurement model; Model 2: Cognitive skills and reading measurement models; Model 3: Cognitive skills and reading structural equation models; GFI: goodness of fit index; RMSEA: root mean square error of approximation; AGFI: adjusted goodness of fit index; NFI: normed fix index; CFI: comparative fit index; IFI: incremental fit index.
